# The Duty of the Organization to the Physician

**Published:** 1915-08

**Authors:** 


### The Duty of the Organization to the Physician.

The converse duty of the physician to affiliate himself with professional organizations may be taken for granted. In fact, such affiliation is not merely a duty but a matter of self-interest except in the very narrow sense, that a very narrow-minded man may be correct in assuming that he will not be able to get anything out of professional associations that he can turn into dollars and cents. The normal individual, however, very frankly recognizes that he learns things at medical societies which both increase his earning power and save him from errors in practice and in conforming to legal restrictions that would be costly.

But, occasionally, it is well to change the point of view and, at present, we want to emphasize the duty of the organization to the man. This point of view is useful as well as philanthropic for a concrete example of service by the society to the individual physician is the best possible argument in winning reluctant physicians into the fold.

Some time ago, we noted a court decision by which a society was compelled to assume the defense of one of its members. We do not know the exact circumstances. Possibly the physician was unpopular; perhaps there was a question as to the merits of the case; the main fact was that the society had tried to squirm out of a duty owed to every member, on a technical point of date and that its attempt was unsuccessful. We imagine that that society does not have anything like 100% of the membership which it logically should have. The anti-narcotic laws are bound to result in many indictments or technicalities. In many other ways, physicians, either entirely without blame or under extenuating circumstances or, at least with the presumption of innocence till proved guilty which is part of the foundation of democracy, are liable to require legal assistance or, at least, the moral support and friendship of their associates. We believe that it is not only the duty but the self-interest of the profession generally, as organized, to support any member who is in trouble. Insurance of legal support cannot very well be extended to cases in which the private business and private conduct of a physician come into question nor can it be expected that warm comradeship will be experienced by the man who is naturally crooked or who has been remiss in his own duties toward his fellows. But no technicality and no lack of influence should stand in the way of a full discharge of the debt of responsibility.

We have also urged that steps should be taken to care for disabled, sick and age-worn members of the profession, to keep them from want and to show the people that the medical profession is not lacking in fraternal spirit. Anything approaching adequate fraternal insurance will involve individual sacrifice and will present many difficult problems in organization and administration. Considerable time will be required to carry out any such scheme. Meantime, we believe that, without direct expense, much may be done that will not only be a comfort to the unfortunate but that will strengthen the profession both in its general influence and in its organization. Some apparently unrelated facts may be very pertinent to this phase of the subject. For example, the best statistics of professional mortality, those of the Journal of the A. M. A., show a death rate of only a little over 14:1000 for the medical profession. Often, in noting the death of an old physician, we find no record of him in medical directories. Often, we learn casually that a business or social acquaintance is a medical graduate. All these facts show that a great many physicians drift from their professional affiliations soon after they retire from active practice, either on account of age or from engaging in other lines of work. Very few, under such circumstances, feel that it is worth while to maintain their dues or to take an active part in professional life, yet we feel that it is both a duty and for the interest of organized medicine to maintain a hold on such men, unless, of course, they have drifted into lines of commercial work directly at variance with the ethical standards of the profession.

Consider, too, the case of the struggling physician who has no notion of abandoning practice permanently but who has been incapacitated for a long period by an accident or by sickness, or who has had to change his residence and endure for a second time and with greater family obligations, the ordeal of gaining a practice. His removal may have been due to failure of health of himself or a member of his family, requiring a different climate; to business reasons, even to mere restlessness and impatience. At any rate, even the comparatively small item of annual dues becomes a relatively large expense and one that is not so imperative as the demand for food, clothing and lodging. In all other respects, he is even more in need of professional affiliation while, save for his inability to contribute financially to the society, he is just as valuable an asset to it as if he were in full practice. Suppose that he fails to get on his feet again, is his opinion of professional organization or that of any disinterested layman who happens to know of the case likely to be favorable? Suppose that he regains his professional footing in a purely commercial sense, is he likely to become a hearty, active, influential supporter of professional organization? Is there not rather danger that he will develop into almost the greatest offset that organized medicine has to deal with—the successful, competent quack?

Some years ago, we were much impressed with a contrast between professional circumstances attending the death of two physicians. Both were, at corresponding ages, good types of average success, professional activity and uprightness of life. One died after a brief illness. A special meeting of the county society was called, appropriate memorials were presented and the society attended his funeral in a body. The other died after several years of invalidism and the profession ignored his departure for he had forfeited his membership for the very simple reason of the greater practical demand for food, clothing and lodging previously mentioned. Save for the naturally greater interest in the sudden death of one in active life, the popular press and the laity paid the same respect to one as to the other. Without any special personal acquaintance with either, we admit frankly that we can understand how the multiplication of such instances can exert a very marked influence in determining the attitude of young and impressionable physicians toward professional organization in general and even with regard to adherence to ethical professional standards or those of the medical Ishmaelite.

It would cost little beyond the postage on announcements of meetings to provide for the retention on the rolls of medical societies of superannuated and retired physicians and for the temporary transference—confidential if desired—of those for the time being in straightened circumstances, to associate membership. There would be a slight loss of dues from those who, in spite of retirement or misfortune, would continue active membership. On the other hand, professional solidarity would be increased. Organized medicine would stand higher in the community and in the profession. Fewer licentiates would develop into unethical practitioners and there would be less difficulty in bringing individuals into organizations. The numeric increase of membership alone would more than compensate for any conceivable expense or loss.

